# Interface Design in Bimetallic PdNi Nanowires for Boosting Alcohol Oxidation Performances

**DOI:** 10.3390/nano15131047

**Published:** 2025-07-05

**Authors:** Zhen He, Huangxu Li, Lingwen Liao

**Affiliations:** 1 Key Laboratory of Materials Physics, Anhui Key Laboratory of Nanomaterials and Nanotechnology, CAS Center for Excellence in Nanoscience, Institute of Solid State Physics, Hefei Institutes of Physics Science, Chinese Academy of Sciences, Hefei 230031, China; zhenhe@cuhk.edu.hk; 2 Department of Applied Physics, The Hong Kong Polytechnic University, Hung Hom, Kowloon 999077, Hong Kong SAR, China; huangxu.li@polyu.edu.hk

**Keywords:** bimetallic nanostructure, Pd nanowires, interface design, strain engineering, alcohol oxidation

## Abstract

The rational design of a bimetallic nanostructure with a phase separation and interface is of great importance to enhance electrocatalytic performance. Herein, PdNi heterostructures with controlled elemental distributions were constructed via a seeded growth strategy. Partially coated Ni islands in the Pd-Ni nanowire and strained Pd branches in the Pd-NiPd nanowires are revealed, respectively. Impressively, Pd-NiPd nanowires with abundant branches exhibit a superior mass current density and cycling stability toward an ethanol oxidation reaction (EOR) and ethylene glycol oxidation reaction (EGOR). The highest mass activities of 8.63 A mg_Pd_^−1^ and 12.53 A mg_Pd_^−1^ for EOR and EGOR, respectively, are realized on the Pd-NiPd nanowires. Theoretical calculations indicate that the Pd (100)-PdNi (111) interface stands out as an active site for enhancing OH adsorption and the decreasing CO bonding interaction. This study not only puts forward a simple method to construct bimetallic nanostructures with desired elemental distributions and interfaces but also demonstrates the significance of interface engineering in regulating the catalytic activity of metallic nanomaterials.

## 1. Introduction

Bimetallic nanostructures, especially alloys, have drawn considerable research interest in the past several decades [1,2,3,4]. This is due to the fact that the incorporation of secondary metals can effectively modulate the lattice and electronic properties of single metals, leading to the improvement in various catalytic reactions [5,6]. However, the random element distributions in the alloys could limit the performance of constituent metals [7,8]. For instance, Huang et al. found that Au-Pd separation leads to enhanced oxidation performances, compared to AuPd alloys. Similar results have been observed in the multi-metallic nanomaterials. In the PtNiCu nanostructures, the phase separation of Pt_3_Ni and Pt_3_NiCu also results in the improvement of electrocatalytic performances towards a methanol oxidation reaction (MOR) and a hydrogen evolution reaction (HER) [8]. However, the rational design of bimetallic nanostructures with desired elemental distributions and interfaces under mild conditions still remains quite challenging.

Pd has been recognized as an effective catalyst in reducing the energy barrier of alcohol oxidation [9,10,11,12]. Specifically, CO and OH are two essential intermediates of alcohol oxidation reactions (AORs) [2,13,14]. The active sites on the surface of Pd mitigate CO poisoning and strengthen OH adsorption, resulting in an excellent mass activity and durability. Numerous efforts have been undertaken to further improve the electrocatalytic property. For instance, Liu et al. reported that the twin structure in the Pd nanoparticles optimized the adsorption interactions toward reaction intermediates during ethanol oxidation reactions (EORs) [9]. Among various strategies, alloying with other metals to form bimetallic nanostructures has attracted broad research attention [12,15,16]. The incorporation of other transition metal elements into Pd can achieve the regulation of the lattice and electronic parameters of Pd. For example, You et al. have prepared PdSn alloys, which exhibit an excellent catalytic performance toward AORs [15]. Nevertheless, in most of the previous studies, few attempts have been made to control the elements’ distributions and interface.

Tuning the lattice strain of nanomaterials has emerged as a promising strategy to regulate their catalytic performances [17,18,19]. Typically, the lattice strain affects the electronic structure of metal catalysts, thereby influencing the absorption interaction of chemical species on the catalyst surface [19]. The optimized electronic band structure results in the superior catalytic performances of the metallic catalysts. The lattice strain of Pd significantly influences the performances of AORs. For instance, Lin et al. revealed the strain-regulated electrocatalytic performance of Pd nanoparticles [20]. The lattice structure of Pd was regulated by the laser ablation. Although numerous studies have demonstrated the importance of lattice strain in enhancing the performances of Pd-based electrocatalysts, it remains a large challenge to develop a simple method to regulate the lattice structure of Pd.

In this study, bimetallic PdNi nanowires have been synthesized through a seeded-mediated growth method. Specifically, Pd-Ni nanowires were formed by the secondary growth of Ni on the Pd nanowires, where partially coated Ni islands were revealed. NiPd branches grown on the Pd nanowires were also synthesized (i.e., Pd-NiPd), which exhibited strained Pd islands and abundant interfaces. When employed as electrocatalysts toward EORs and ethylene glycol oxidation reactions (EGORs), Pd-NiPd nanowires demonstrate a superior mass current density and cycling stability, outperforming those of Pd-Ni nanowires, Pd nanowires, and commercial Pd/C catalysts. The highest mass activities of 8.63 A mg_Pd_^−1^ and 12.53 A mg_Pd_^−1^ for the EOR and EGOR, respectively, were realized on the Pd-NiPd nanowires. This work could provide opportunities for the rational construction of heterometallic nanostructures for many catalytic reactions.

## 2. Materials and Methods

### 2.1. Materials Synthesis

To synthesize Pd-Ni and Pd-NiPd nanowires, Pd nanowires were firstly prepared based on previous work [10,11]. Typically, 900 mg poly(vinylpyrrolidone) (Sigma-Aldrich, St. Louis, MO, USA), 0.12 mmol PdCl_2_ (Sigma-Aldrich, St. Louis, MO, USA), and 2.0 mmol NaI (Sigma-Aldrich, St. Louis, MO, USA) were dissolved in 15.0 mL deionized water. The mixture was transferred to a 50 mL autoclave, which was then heated to 210 °C. After heating for 15 min, the mixture was maintained at 210 °C, lasting for 4 h. The final products were washed with ethanol and isopropanol and dispersed into 5.0 mL ethanol for further usage. The concentration of Pd was measured to be 0.17 mg/mL by Inductively Coupled Plasma Optical Emission Spectrometer (PerkinElmer, Waltham, MA, USA). The Pd nanowires serve as seeds for secondary growth of Ni to form Pd-Ni nanowires. Then, 6.0 mg Ni (acac)_2_ (Sigma-Aldrich, St. Louis, MO, USA) and 5.0 mg n-hexadecyltrimethylammonium chloride (Sigma-Aldrich, St. Louis, MO, USA) was dissolved in 8.0 mL oleylamine (Sigma-Aldrich, St. Louis, MO, USA) and was added into a 40 mL glass bottle, containing 2.0 mL of Pd nanowires. The mixture was bubbled with N_2_ for 30 min to remove solvent and then ultrasonicated for 15 min. The homogeneous mixture was heated to 150 °C. After heating for 8 min, the mixture was maintained at 150 °C, lasting for 8 h. Ultimately, the Pd-Ni nanowires were washed with ethanol and hexane and dispersed into 1.0 mL ethanol for further usage.

Pd-NiPd nanowires were prepared via nucleation and growth of Pd on the Pd-Ni nanowires. Specifically, 1.0 mg Pd(acac)_2_ (Sigma-Aldrich, St. Louis, MO, USA) and 3.0 mg tetraoctylammonium bromide (Sigma-Aldrich, St. Louis, MO, USA) were dissolved in 3.0 mL oleylamine, which was treated by ultrasound to ensure full dissolution. After that, 0.5 mL of Pd-Ni nanowires dispersed in ethanol was transferred into the mixture, which was bubbled with N_2_ for 30 min to remove the ethanol. Finally, the mixture was heated to 100 °C. After heating for 5 min, the mixture was maintained at 100 °C, lasting for 5 h. Pd-NiPd nanowires were obtained after the products were washed over three times with ethanol and hexane.

### 2.2. Materials Characterization

The morphology of Pd-Ni nanowires and Pd-NiPd nanowires was characterized by the transmission electron microscopy (TEM) and scanning TEM (STEM) on a JEOL JEM-2100F microscope (JEOL, Tokyo Metropolis, Japan). During the operation of microscope, molybdenum meshes were used and the voltage was 200 kV. A JEOL ARM200F (JEOL, Tokyo Metropolis, Japan) running at 200 kV with a chilled FEG provides high-angle annular dark-field (DF) STEM photos. To reveal the chemical states of Pd and Ni, a Kratos AXIS Supra (Shimadzu, Manchester, UK) has been employed. C 1s spectrum with a standard binding energy of 284.8 eV was used to modify the binding energy. To demonstrate the detailed elemental concentrations of Pd and Ni, the Optima 8000 DV equipment (PerkinElmer, Waltham, MA, USA) was deployed.

### 2.3. Electrochemical Measurements

Electrochemical ethanol oxidation reaction (EOR) and ethylene glycol oxidation reaction (EGOR) were tested under alkaline medium [21,22]. The catalysts were loaded on the working electrode (glassy carbon). Before measurements, carbon-supported bimetallic PdNi catalysts were firstly prepared. Specifically, the catalyst that contains 100 µg Pd was dropwisely added to the carbon suspension that contains 900 µg carbon black. After thorough sonication for 1 h, 10 µL Nafion solution was dropped into the mixture. The final homogeneous mixture was dropped on the working electrode, resulting in an active metal loading of 5.1 µg cm^−2^. Pt and Hg/HgO worked as counter and reference electrodes, respectively. The reference electrode was calibrated to the reversible hydrogen electrode. The experiments were carried out on the CHI 760E workstation (Chenhua, Shanghai, China). During the electrochemical test, the cyclic voltammetry (CV) curves were recorded from 0.02 to 1.22 V (vs. reversible hydrogen electrode). The electrolytes were 1.0 M KOH solution containing 1.0 M ethanol or ethylene glycol under a nitrogen atmosphere.

### 2.4. DFT Calculations

The density functional theory (DFT) calculation was carried out by employing previously reported method [23,24,25]. Specifically, the adsorption energies of various reaction intermediates were acquired based on the Vienna Ab Initio Simulation Package (VASP) (University of Vienna, Vienna, Austria). The exchange–correlation interactions were described with the Perdew–Burke–Ernzerhof (PBE) function [23]. A plane-wave energy cutoff of 500 eV was applied, with energy and force convergence criteria set to 1.0 × 10^−5^ eV per atom and −0.02 eV Å^−1^, respectively. Parameter SIGMA and ISMEAR were set to 0.2 and 1, respectively.

## 3. Results and Discussion

**Figure 1:**
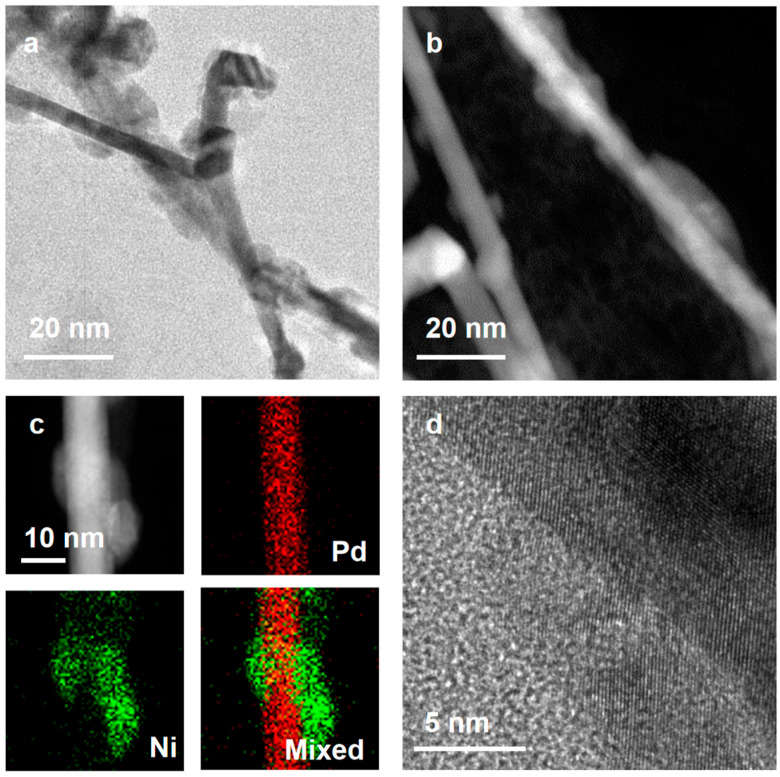
The characterization of Pd-Ni nanowires. (**a**) The TEM image of Pd-Ni nanowires. (**b**) The STEM image of the Pd-Ni nanowire. (**c**) The STEM image and the corresponding elemental mappings of a representative Pd-Ni nanowire. (**d**) The high-resolution TEM image of a typical Pd-Ni nanowire.

The Pd-Ni nanowires were prepared through the seeded growth on the pre-designed Pd nanowires [10,11]. Pd nanowires, as templates, were firstly synthesized based on previous research. As shown in Figure S1, uniform Pd nanowires were prepared with an average diameter of 6.2 nm. Pd-Ni nanowires were then constructed through the reduction and growth of the Ni species on the Pd surface. Figure 1a,b show the TEM and scanning TEM (STEM) images of Pd-Ni nanowires, respectively. Ni islands were partially coated on the Pd nanowires, which may originate from the large lattice mismatch between Pd and Ni elements. The huge strain between the Pd and Ni regulates the growth model, resulting in the anisotropic growth of bimetallic heterostructures [26]. To further affirm the elemental distributions of Pd and Ni, STEM energy-dispersive X-ray spectroscopy (EDS) was employed. As shown in Figure 1c, Ni elements were not uniformly distributed on the Pd nanowire. The atomic structure of bimetallic Pd-Ni nanowires was explored by the atomic-scale TEM (Figure 1d). The continuous lattice fringes from the Pd nanowire to the Ni islands indicate the epitaxial growth behavior. The lattice distance of the Ni (111) planes was calculated as 0.21 nm, which shows a 5% expansion compared to that of the standard Ni (111). The lattice expansion could be related to the larger lattice parameter of the Pd seed. The atomic contents were measured by the EDS (Figure S2), showing an atomic ratio of Pd_39.8_Ni_60.2_.

**Figure 2:**
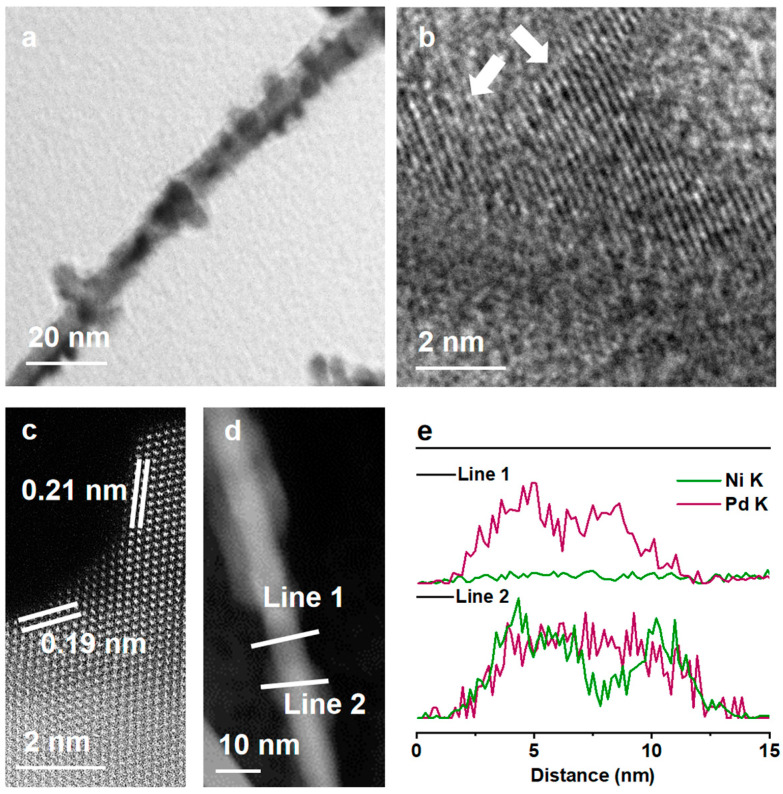
Characterization of Pd-NiPd nanowires. (**a**) TEM image. (**b**) High-resolution TEM image. (**c**) Atomic-resolution HAADF-STEM image. (**d**) STEM image. (**e**) Line scans in d.

The as-designed Pd-Ni nanowires then worked as seeds for the secondary growth of Pd to form the Pd-NiPd heterostructure. Figure 2a and Figure S3 demonstrate the architecture of the Pd-NiPd nanowires. There are multiple tiny NiPd islands grown on the Pd nanowires. During the growth of the Pd, the reaction temperature should be carefully controlled to avoid the self-nucleation of Pd nanocrystals (Figure S4). The crystal structures of Pd-NiPd nanowires were further investigated. Figure 2b shows the interface on the Pd nanowires. The high-angle annular dark-field (HAADF) STEM was utilized to attain the atomic structure. As exhibited in Figure 2c, an interplanar spacing of 0.19 nm on the surface of the Pd nanowire could be attributed to the Pd (100) facet, while the (111) facet on the island shows a lattice distance of 0.21 nm, showing a lattice compression of 4.5% in comparison with that of the standard Pd (111) facet. The EDS line scans were used to quantitatively explore their elemental distributions. As shown in Figure 2d,e, there are negligible amounts of Ni along the radius direction across line 1. However, along line 2, PdNi was revealed, indicating the formation of bimetallic branches on the Pd nanowire. The formation of PdNi may originate from the Galvanic Replacement reaction between the Ni and Pd, considering that the standard reduction potential of Pd (+0.951 V) is higher than that of Ni (−0.250 V). The uneven distribution of the Ni coating on the Pd provides an opportunity to realize anisotropic element distributions and the Pd (100)-PdNi (111) interface design in the bimetallic nanowires. The overall atomic ratio was measured as Pd_53.4_Ni_46.6_ based on the EDS results (Figure S5).

**Figure 3:**
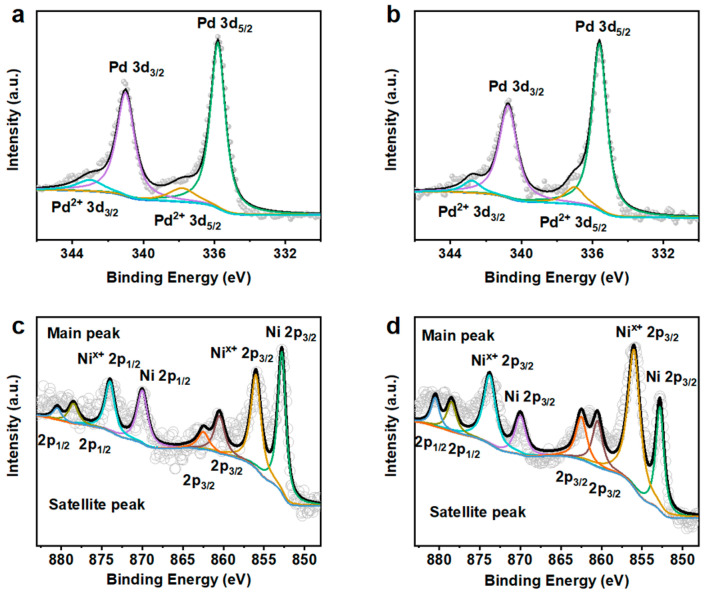
XPS characterization of Pd-Ni and Pd-NiPd nanowires. (**a**) High-resolution Pd 3d spectrum of Pd-Ni nanowires. (**b**) High-resolution Pd 3d spectrum of Pd-NiPd nanowires. (**c**) High-resolution Ni 2p spectrum of Pd-Ni nanowires. (**d**) High-resolution Ni 2p spectrum of Pd-NiPd nanowires.

X-ray photoelectron spectroscopy (XPS) was used to analyze the electronic structures of Pd and Ni in the Pd-Ni and Pd-NiPd nanowires. Figure 3a,b show the high-resolution Pd 3d spectrum, affirming that Pd was significant in the metallic state in two samples. Minor Pd ^2+^ peaks were also detected, which may correlate with the slight surface oxidation or electron transfer interaction [20]. Importantly, compared with those of Pd-Ni nanowires (Figure 3a), the binding energy of Pd 3d_5/2_ in Pd-NiPd nanowires exhibits a negative shift (~0.2 eV). The reduced binding energy could originate from the strained Pd branches [19], which was consistent with the TEM results. Figure 3c,d show the high-resolution Ni 2p spectrum in Pd-Ni and Pd-NiPd nanowires, respectively. The Ni 2p spectrum demonstrates main peaks and satellite peaks. Inevitable oxidation peaks were revealed, which is attributed to the reactivity of Ni on its exposure to air [27]. The atomic ratios of Pd and Ni on the Pd-Ni nanowires and Pd-NiPd nanowires were calculated as 32.1/67.9 and 43.2/56.8, respectively. These results are lower than those of the EDS results, which may be attributed to the Ni-riched surfaces.

**Figure 4:**
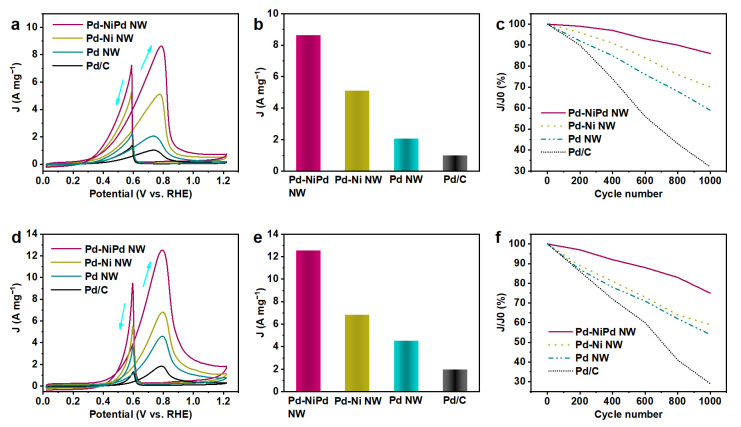
The electrocatalytic property. (**a**) Pd mass-normalized CV curves of the commercial Pd/C, Pd nanowire (NW), Pd-Ni NW, and Pd-NiPd NW in a N_2_-saturated 1.0 M KOH aqueous solution containing 1.0 M CH_3_CH_2_OH at a scan rate of 50 mV s^−1^. (**b**) Mass activities of the catalysts at their corresponding peak potentials in the CV curves in (**a**). (**c**) The long-term cycling stability retention curves in 1.0 M CH_3_CH_2_OH, where J0 is the initial current density and J is the current density after varying the number of turns. (**d**) Pd mass-normalized CV curves of the commercial Pd/C, Pd nanowire (NW), Pd-Ni NW, and Pd-NiPd NW in a N_2_-saturated 1.0 M KOH aqueous solution containing 1.0 M ethylene glycol at a scan rate of 50 mV s^−1^. (**e**) Mass activities of the catalysts at their corresponding peak potentials in the CV curves in (**d**). (**f**) The long-term cycling stability retention curves containing 1.0 M ethylene glycol.

The prepared Pd-Ni and Pd-NiPd nanowires were tested as catalysts for electrochemical alcohol oxidation, including the EOR and EGOR. As a comparison, Pd nanowires and commercial Pd/C (10 wt.% loading) were also tested. Figure 4a shows the CV curves recorded in 1.0 M KOH containing 1.0 M ethanol. The Pd-NiPd nanowires demonstrated the highest current density from 0.4 V to 1.2 V (vs. the reversible hydrogen electrode, RHE). The arrow pointing to the positive direction represents the peak that sweeps from the lower to higher potential (vice versa). Pd nanowires and Pd-Ni nanowires also exhibit a superior mass current compared to commercial Pd/C catalysts. Their mass activities calculated at corresponding peak potentials were obtained (Figure 4b). Pd-NiPd nanowires exhibit the greatest current density of 8.63 A mg_Pd_^−1^, which is 1.7, 4.2, and 8.5 times higher than those of the Pd-Ni nanowires, Pd nanowires, and commercial Pd/C, respectively. Apart from the oxidation activity, cycling stability stands out as another important factor in measuring the EOR performance. Figure 4c exhibits the cycling stability of Pd-NiPd nanowires, Pd-Ni nanowires, Pd nanowires, and Pd/C catalysts. In the initial 200 cycles, a slight decrease in mass activity was revealed on the Pd-NiPd nanowires. The current density was still maintained at over 85% after 1000 cycles. As a comparison, the cycling performances of Pd-Ni nanowires and Pd nanowires were less satisfactory. Only 30% of current density remained on the commercial Pd/C catalyst after 1000 cycles. These results suggest that Pd-NiPd nanowires exhibit an enhanced activity and stability toward EORs. The performance of the Pd-NiPd catalyst was better than many previously reported electrocatalysts for EORs (Table S1) [28]. In addition, Fourier transform infrared spectroscopy (FT-IR) was used to observe the reaction intermediates on the Pd-NiPd nanowires (Figure S6). A strong band at ~1645 cm^−1^ may originate from the adsorbed water, which prompts the formation of OH* [8,29]. Almost no CO peak located at ~2100 cm^−1^ was detected, indicating that CO* was effectively removed.

In addition to the EOR, EGOR performances of four catalysts were also evaluated based on the similar procedure. Figure 4d exhibits the CV curves of Pd-NiPd nanowires, Pd-Ni nanowires, Pd nanowires, and Pd/C catalysts. The highest mass activity of 12.53 A mg_Pd_^−1^ was realized on the Pd-NiPd nanowires (Figure 4e), which is much greater than those of Pd-Ni nanowires (6.82 A mg_Pd_^−1^), Pd nanowires (4.53 A mg_Pd_^−1^), and Pd/C (1.85 A mg_Pd_^−1^). With regard to the cycling stability (Figure 4f), nearly 80% of the current density remained after 1000 cycles on the Pd-NiPd nanowires, outperforming the Pd-Ni nanowires (59%), Pd nanowires (54%), and Pd/C (29%). The performance of the Pd-NiPd catalyst was better than many previously reported electrocatalysts for EGORs (Table S2) [28]. Diverse C1 and C2 intermediates were detected during the EGOR, based on the FT-IR result (Figure S7). The aforementioned data indicated that Pd-NiPd nanowires work as highly efficient catalysts toward AORs.

**Figure 5:**
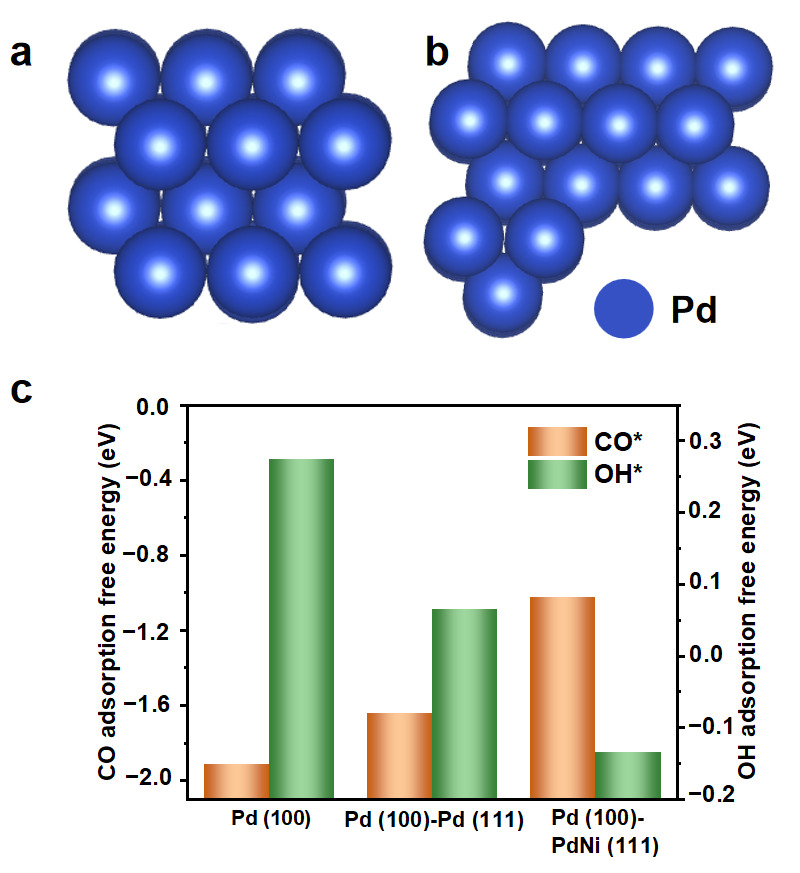
DFT calculations. (**a**,**b**) The atomic model of the Pd (100) surface and Pd (100)-Pd (111) interface. (**c**) Adsorption free energies of CO and OH on the active sites of the Pd (100), Pd (100)-Pd (111) interface, and Pd (100)-PdNi (111) interface.

During the AOR, CO* and OH* are essential reaction intermediates [9,30]. The CO* bonded on the surface of the catalyst could cause poisoning effects, leading to poor activity and stability. Contrary to the CO* intermediate, appropriately enhancing the OH* adsorption is desirable, considering that OH* can prompt the oxidation reaction of CO* [14]. Therefore, a density functional theory (DFT) calculation was carried out to understand the bonding interaction of CO* and OH* on the bimetallic PdNi nanowires (Figure 5a,b). Compared with the Pd (100) and Pd (100)-Pd (111) interface, the Pd (100)-PdNi (111) interface on the Pd-NiPd nanowires stands out as an effective site (Figure 5c). The lattice strain along the 111> direction was modeled by incorporating Ni into the Pd lattice and replacing the Pd (111) facet with the PdNi alloy (111) facet. The high CO* adsorption energy on the Pd (100)-PdNi (111) interface indicates the weakened bonding interaction toward CO* during the alcohol oxidation. Moreover, the lowest adsorption energy toward OH* was also revealed, which is highly desirable. The theoretical calculation results further affirm the superior electrochemical property of Pd-NiPd nanowires.

## 4. Conclusions

In conclusion, PdNi bimetallic nanostructures with a desired components separation and interfaces were constructed via a seeded growth strategy. Partially coated Ni islands in the Pd-Ni nanowire and strained Pd branches in the Pd-NiPd nanowire were revealed, respectively. Compared with Pd-Ni nanowires, Pd nanowires, and commercial Pd/C catalysts, Pd-NiPd nanowires with abundant branches exhibited a superior mass current density and cycling stability toward EORs and EGORs. The highest mass activities of 8.63 A mg_Pd_^−1^ and 12.53 A mg_Pd_^−1^ for the EOR and EGOR, respectively, were realized on the Pd-NiPd nanowires. DFT calculations indicated that the Pd (100)-PdNi (111) interface stands out as an active site for enhancing OH adsorption and decreasing CO bonding interactions. This work demonstrates the importance of the elemental distribution, interface design, and strain engineering of metallic catalysts for alcohol oxidation.

## Data Availability

The original data are included in the article/Supplementary Materials, and further inquiries can be directed to the corresponding author.

## Supplementary Materials

The following supporting information can be downloaded at https://www.mdpi.com/article/10.3390/nano15131047/s1. Figure S1: TEM image of Pd nanowires. Figure S2: EDS spectrum of Pd-Ni nanowires. Figure S3: TEM image of Pd-NiPd nanowires. Figure S4: TEM image of self-nucleation Pd nanocrystal when the reaction temperature for Pd-NiPd increased to 120 ℃. Figure S5: EDS spectrum of Pd-NiPd nanowires. Figure S6: The FT-IR result of EOR on the Pd-NiPd nanowires. Figure S7: FT-IR result of EGOR on the Pd-NiPd nanowires. Table S1: Comparison of the electrocatalytic performances of Pd-based catalysts toward EOR in some recently reported representative works and this work. Table S2: Comparison of the electrocatalytic performances of Pd-based catalysts toward EGOR in some recently reported representative works and this work.
