# Supplementary material for: Interface Design in Bimetallic PdNi Nanowires for Boosting Alcohol Oxidation Performances
Source: Nanomaterials (Basel). 2025 Jul 5;15(13):1047. doi: 10.3390/nano15131047 (PMC12250756; doi:10.3390/nano15131047)
Supplement: Supplementary file 1 [file nanomaterials-15-01047-s001.zip › nanomaterials-3682628-supplementary.pdf]

Interface Design in Bimetallic PdNi Nanowires for Boosting Alcohol Oxidation Performances

Zhen He [1], Huangxu Li [2] and Lingwen Liao [1,*]

[1]Key Laboratory of Materials Physics, Anhui Key Laboratory of Nanomaterials and Nanotechnology, CAS Center for Excellence in Nanoscience, Institute of Solid State Physics, HFIPS, Chinese Academy of Sciences, Hefei 230031, China

[2]Department of Applied Physics, The Hong Kong Polytechnic University, Hung Hom, Kowloon 999077, Hong Kong SAR, China

*Corresponding author

E-mail: liaolw@issp.ac.cn

Supplementary Figures

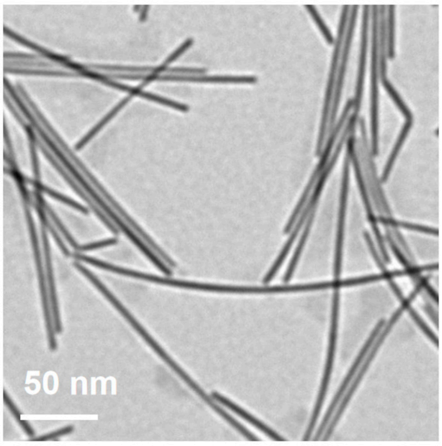

Figure S1. TEM image of Pd nanowires.

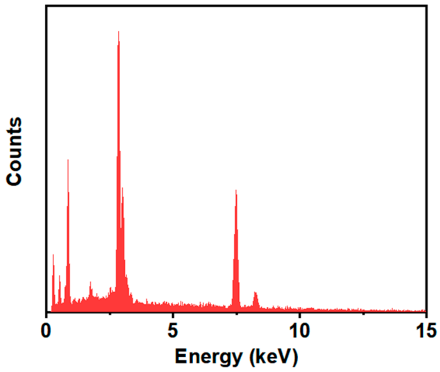

Figure S2. EDS spectrum of Pd-Ni nanowires.

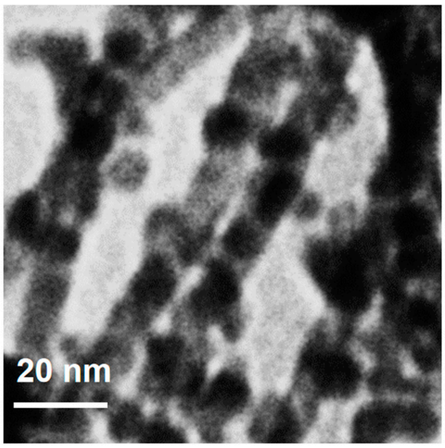

Figure S3. TEM image of Pd-NiPd nanowires.

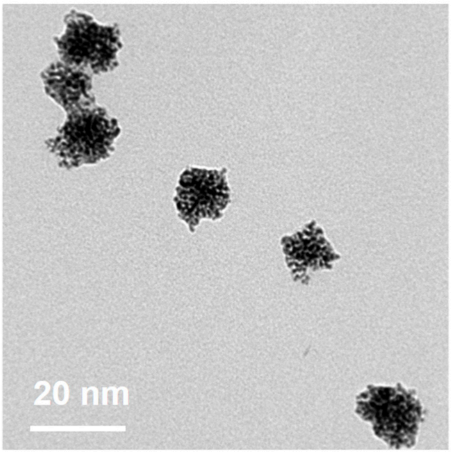

Figure S4. TEM image of self-nucleation Pd nanocrystal when the reaction temperature for Pd-NiPd increased to 120 °C.

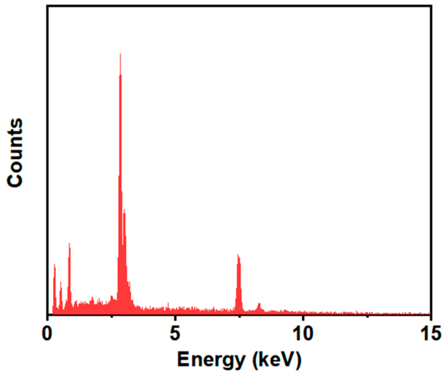

Figure S5. EDS spectrum of Pd-NiPd nanowires.

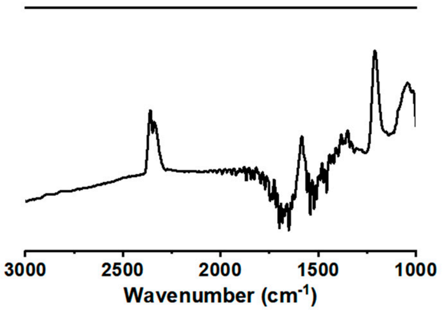

Figure S6. The FT-IR result of EOR on the Pd-NiPd nanowires.

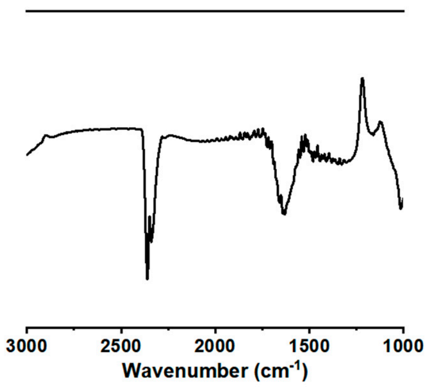

Figure S7. FT-IR result of EGOR on the Pd-NiPd nanowires.

Supplementary Tables

Table S1. Comparison of the electrocatalytic performances of Pd-based catalysts toward EOR in some recently reported representative works and this work.

Catalyst	Electrolyte	Mass activity ($A\ mg^{-1}$)	Ref.
**Pd-NiPd nanowires**	**1.0 M KOH + 1.0 M ethanol**	**8.63**	**This work**
Pd/N-doped graphene	1.0 M KOH + 1.0 M ethanol	2.69	Appl. Catal. B 2021, 280, 119464
PdAg worm-like networks	1.0 M KOH + 1.0 M ethanol	3.48	J. Colloid Interf Sci. 2020, 574, 182
Pd nanowire @ CuOx	1.0 M KOH + 1.0 M ethanol	0.55	Small 2020, 16, 1904964
PdAgCu nanospheres	1.0 M KOH + 1.0 M ethanol	4.64	Chem. Sci. 2019, 10, 1986
Mesoporous Pd film	1.0 M KOH + 1.0 M ethanol	0.66	Nat. Protoc. 2020, 15, 2980
PdBi-$Bi(OH)_3$ nanochains	1.0 M NaOH + 1.0 M ethanol	5.30	Nano Lett. 2019, 19, 4752
Twin-riched Pd	1.0 M KOH + 1.0 M ethanol	1.85	Adv. Energy Mater. 2022, 12, 2103505
Stepped Pd nanowires	1.0 M KOH + 1.0 M ethanol	6.38	J. Colloid Interf Sci. 2023, 646, 529
PdNiP/C	1.0 M KOH + 1.0 M ethanol	0.95	J. Energy Chem. 2021, 58, 256

Table S2. Comparison of the electrocatalytic performances of Pd-based catalysts toward EGOR in some recently reported representative works and this work.

Catalyst	Electrolyte	Mass activity (A mg^{-1})	Ref.
**Pd-NiPd nanowires**	**1.0 M KOH + 1.0 M EG**	**12.53**	**This work**
Stepped Pd nanowires	1.0 M KOH + 1.0 M EG	7.98	J. Colloid Interf Sci. 2023, 646, 529
PdNiP/C	1.0 M KOH + 1.0 M EG	3.10	J. Energy Chem. 2021, 58, 256
PdAg nanoparticles	1.0 M KOH + 1.0 M EG	5.33	J. Colloid Interface Sci. 2019, 544, 284
Branched PdCu alloy	1.0 M KOH + 0.5 M EG	1.65	Chem. Commun. 2018, 54, 13363
PdCuTe nanowires	1.0 M KOH + 1.0 M EG	3.87	Nano Res. 2019, 12, 351
Concave PdRu nanocubes	1.0 M KOH + 1.0 M EG	3.35	Appl. Surf. Sci. 2018, 427, 83
